# Acupuncture for the treatment of overactive bladder: A systematic review and meta-analysis

**DOI:** 10.3389/fneur.2022.985288

**Published:** 2023-01-12

**Authors:** Jung-Ju Lee, Jeong-Weon Heo, Tae-Young Choi, Ji Hee Jun, Myeong Soo Lee, Jong-In Kim

**Affiliations:** ^1^Department of Clinical Korean Medicine, Graduate School, Kyung Hee University, Seoul, Republic of Korea; ^2^KM Science Research Division, Korea Institute of Oriental Medicine, Daejeon, Republic of Korea

**Keywords:** acupuncture, overactive bladder, systematic review, meta-analysis, grade

## Abstract

**Background:**

Acupuncture (AT) successfully regulates overactive bladder (OAB) symptoms. However, previous systematic reviews and meta-analyses have not provided sufficient evidence. This review presents the current evidence of the efficacy of AT in the management of OAB symptoms.

**Methods and analyses:**

A total of 12 databases were searched from their inception: PubMed, EMBASE, Cochrane Central Register of Controlled Trials (CENTRAL), and AMED databases; five Korean medical databases; and three Chinese medical databases. Study selection, data extraction, and assessment were independently performed by two researchers. The risk of bias was assessed using the Cochrane risk of bias assessment tool. RevMan 5.4.1 software was used for data aggregation, and the Grades of Recommendations, Assessment, Development and Evaluation (GRADE) assessment was used to evaluate the quality of the study outcomes.

**Results:**

A total of 30 studies were included in this review. Compared with the sham AT group, the AT group exhibited significant effects in reducing overactive bladder symptom scores (OABSS) [mean difference (MD): −1.13, 95% confidence interval (CI): −2.01 to −0.26, *p* = 0.01 *I*^2^ = 67%] and urinary frequency [standardized mean difference (SMD): −0.35, 95% CI: −0.62 to −0.08, *I*^2^ = 0%]. The AT group showed an equivalent effect as drug therapy in reducing OABSS (MD: −0.39, 95% CI: – 1.92 to 1.13, *p* = 0.61, *I*^2^ = 94%) and urinary frequency (MD: 0.74, 95% CI: −0.00 to 1.48, *p* = 0.05, *I*^2^ = 71%) with fewer adverse events [risk ratio (RR): 0.38, 95% CI: 0.16–0.92, *p* = 0.03, I^2^ = 58%]. The AT plus drug therapy group had a more favorable effect than drug therapy alone for reducing OABSS (MD: −2.28, 95% CI: −3.25 to −1.31, *p* < 0.00001, *I*^2^ = 84%) and urinary frequency (MD: −2.34, 95% CI: −3.29 to −1.38, *p* < 0.00001, *I*^2^ = 88%). The GRADE assessment demonstrated that the level of evidence was mostly low or very low given the high risk of bias and small sample sizes.

**Conclusion:**

AT had more favorable effects than sham AT in reducing OAB symptoms. AT improved OAB symptoms as effectively as conventional drug therapy, and the combination of AT and drug therapy had more favorable effects than drug therapy alone. However, more rigorous studies are needed to enhance the level of evidence.

**Systematic review registration:**

http://www.crd.york.ac.uk/PROSPERO/display_record.php?ID=CRD42014010377, identifier: PROSPERO [CRD42014010377].

## Introduction

Overactive bladder (OAB) refers to urinary urgency that is accompanied by increased frequency and nocturia with or without urgency urinary incontinence in the absence of urinary tract infection (UTI) or other obvious pathology (1). The symptoms of OAB are due to involuntary contractions of the detrusor muscle during the filling phase of the micturition cycle, so-called detrusor overactivity (1). However, only 64% of patients with OAB have uro-dynamically proven detrusor overactivity. Therefore, OAB is a syndrome characterized as a “symptom-based diagnosis” (2).

Once a diagnosis of OAB has been made, most patients progress through a stepwise treatment path from conservative options to medical treatments and finally surgical treatments. As a first step, conservative management includes modifying behaviors and interventions, such as pelvic muscle exercises (3). The second step, pharmacotherapy, employs antimuscarinic agents, which have an antagonistic action on muscarinic receptors throughout the body, therefore affecting both involuntary detrusor contraction and increased sensory afferent signaling. However, despite these effects, antimuscarinic agents have several uncomfortable side effects resulting in an overall poor adherence profile with 17–35% of patients still taking their prescribed drug after 1 year (4). Dry mouth is the most common side effect, and other symptoms, such as blurred vision, constipation, erythema, fatigue, increased sweating, nausea, and vomiting, have also been reported (5). The third step, which includes surgical treatments, such as neuromodulation or botulinum toxin injection, is more invasive; therefore, patients with those conditions may seek other treatment options (6).

The mechanisms underlying acupuncture (AT) for neuromodulation of the bladder are not precisely understood. However, urodynamic evidence of detrusor overactivity suggests that AT suppresses uninhibited bladder contractions (7). Furthermore, AT stimulation seems to pass information *via* sensory ganglia to the spinal cord and *via* interneurons to modulate the activity of motor neurons in the brainstem that controls autonomic function, including urogenital activity, such as detrusor and sphincter muscle activity (8). These findings suggest that AT may help to improve OAB symptoms.

This review aims to systematically evaluate the evidence for the safety and effectiveness of AT for patients with OAB from randomized controlled trials (RCTs).

## Methods

This protocol was registered with The International Prospective Register of Systematic Reviews (PROSPERO) (CRD42014010377). The reporting of this review adheres to the recommendations of the Preferred Reporting Items for Systematic Reviews and Meta-Analyses (PRISMA) (9).

### Search strategy

Electronic databases, including MEDLINE, EMBASE, the Cochrane Central Register of Controlled Trials (CENTRAL), AMED, five Korean databases [KoreaMed, the Korean Traditional Knowledge Portal (KTKP), DBpia, the Research Information Service System (RISS), and the Korean Studies Information Service System (KISS)], and three Chinese databases [China National Knowledge Infrastructure (CNKI), the Chongqing VIP Chinese Science and Technology Periodical Database (VIP), and the Wanfang Database], were searched from their inception to February 2022. Our search strategy included keywords, such as “acupuncture,” “overactive bladder,” “detrusor instability,” and “urinary urgency,” in English, Chinese, and Korean. The search terms for each database are listed in Supplementary material 1.

### Inclusion and exclusion criteria

#### Types of studies

Only RCTs were included in this systematic review. We excluded trials, case studies, case series, qualitative studies, and uncontrolled trials. Trials that failed to provide detailed results were also excluded. RCTs published in the form of abstracts were included. No language restrictions were imposed.

#### Types of participants

We included studies that involved patients with OAB regardless of age, sex, and race. We excluded OAB studies that involved patients with neurological disease, for example, OAB in Parkinson's disease or stroke.

#### Types of interventions and controls

Studies evaluating all types of AT with and without electrical stimulation were included. Studies were included if AT was used as the only intervention. We also included trials in which the control group received general conventional care, such as a behavioral approach, conventional drug treatments, and sham AT (interventions mimicking “true” AT/true treatment). The acceptability of sham AT as a valid control is highly controversial (10, 11), and we planned to analyze the results using subgroup analysis. Pragmatic trials that compared AT with any other treatments (e.g., conventional drugs/exercise and education) were included. Studies were excluded if the AT was a part of a complex intervention. We excluded warm needle AT and fire AT. Studies investigating other methods of AT point stimulation without needle insertion [e.g., acupressure, transcutaneous electrical nerve stimulation (TENS), pressed studs, and laser stimulation] were excluded. Trials were excluded if the study design did not allow for the evaluation of the effectiveness of AT (e.g., use of a treatment with unproven efficacy in the control group or a comparison between two different forms of AT) or if the study adopted comparisons between treatments or groups that were expected to have similar effects to AT (e.g., moxibustion).

#### Types of outcome measures

##### Primary outcome measures

- OABSS: overactive bladder symptom scores.
- Frequency: number of daily urinary events.

##### Secondary outcome measures

- Incontinence: number of daily incontinence events.
- Response rate: number of patients whose OAB symptoms improved/total participants.
- Adverse events (AEs).

### Data collection, extraction, and assessment

#### Study selection

**Figure 1:**
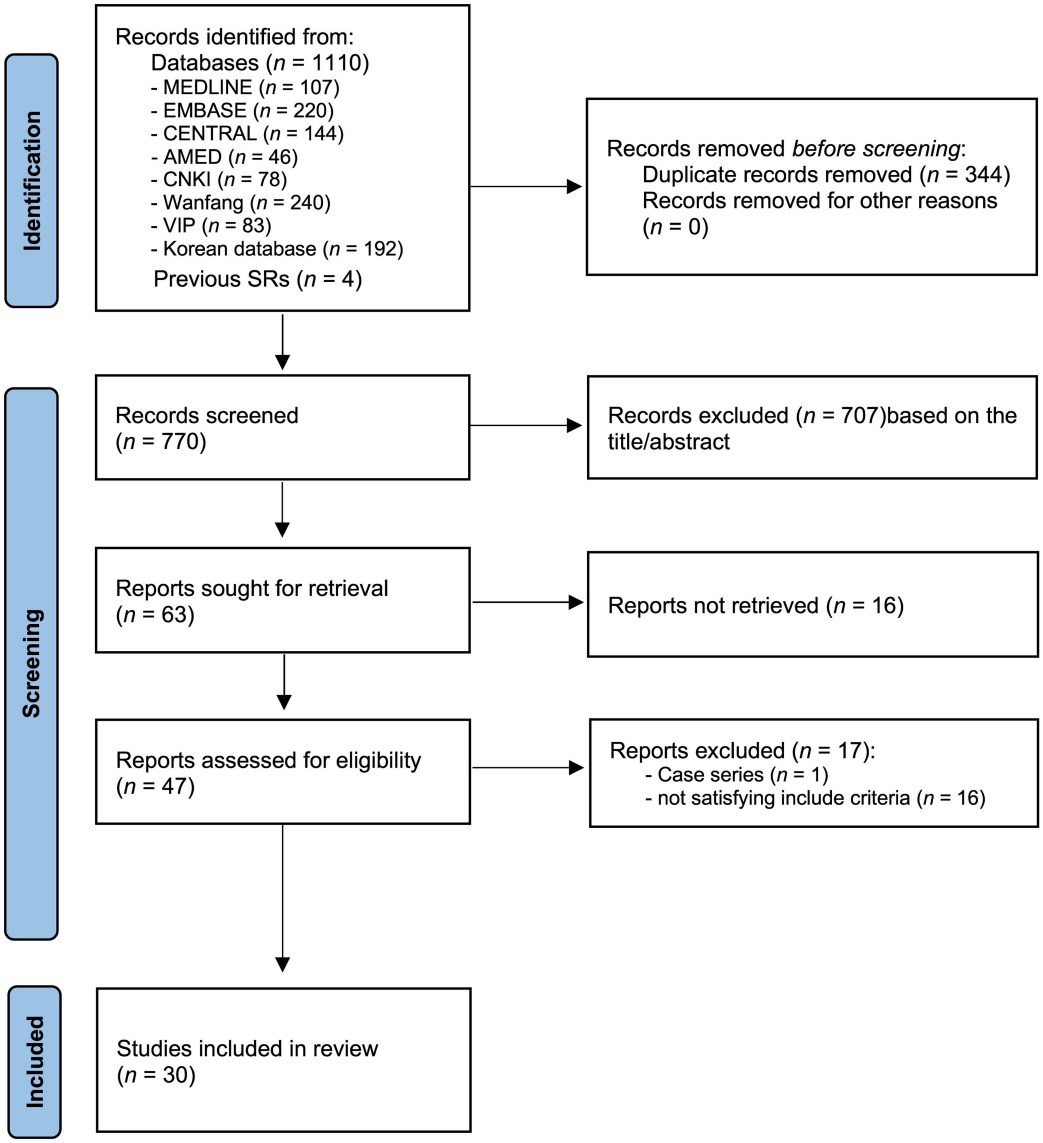
PRISMA 2020 flow diagram for the included studies.

Two reviewers (J-JL and J-WH) independently screened the titles and abstracts of the studies identified in the search, assessed the criteria for study selection, and recorded their decisions based on predefined criteria. Another reviewer (J-IK) resolved any disagreements in the study selection. The study selection process was documented and summarized in a PRISMA flow diagram. The process is presented in Figure 1.

### Data extraction

All articles were read by two independent reviewers (J-JL and J-WH), who extracted data from the articles according to predefined criteria. The extracted data included the author's name(s), year of publication, sample size, age, sex, OAB duration, AT intervention, control intervention, main outcomes, adverse effects, and authors' conclusion. For the extraction of intervention-related information, the revised Standards for Reporting Interventions in Clinical Trials of Acupuncture (STRICTA) items were used to describe the details of AT treatments used in each study (12). When the reported data were insufficient or unclear, the author contacted the first author or corresponding authors by e-mail or telephone to request missing data or clarify data.

### Assessment of risk of bias

Two authors (J-JL and J-WH) independently extracted the data from the included trials. The Cochrane risk of bias tool (13) was used to assess the internal validity of each study. The following characteristics were assessed: (1) random sequence generation, (2) allocation concealment, (3) the blinding of participants and personnel, (4) the blinding of outcome assessment, (5) incomplete outcome data, (6) selective outcome reporting, and (7) other sources of bias (we evaluated baseline imbalance). This review uses “L, U, and H” as keys for these evaluations, where “L” (low) indicates a low risk of bias, “U” (unclear) indicates that the risk of bias is unclear, and “H” (high) indicates a high risk of bias. Disagreements were resolved by discussion among all authors. Information regarding the risk of bias assessment for the included studies is presented in a table, and the results and implications are critically discussed.

### Grades of recommendation, assessment, development and evaluation evaluation

We used the Grades of Recommendation, Assessment, Development and Evaluation (GRADE) system to evaluate the level of evidence (14). We initially gave four points to each outcome in each RCT and then lowered the total scores for defects in bias risks, inconsistency, indirectness, inaccuracy, and publication bias. Bias risks included erroneous randomization methods, absence of allocation concealment, insufficient blinding, and excessive data loss to follow-up. The inconsistencies mainly involved different interventions or evaluation techniques. Indirectness principally included two categories: (1) lack of direct comparison between the two groups and (2) outcome measure sensitivity to direct evaluation of efficacy. Inaccuracy was mainly evaluated based on the width of the confidence interval (CI). Publication bias was related to an unpublished study (usually containing negative results) by the investigator. The quality of evidence was categorized as high, moderate, low, or very low quality.

### Data analysis

All statistical analyses were conducted by the Cochrane Collaboration's software Review Manager (RevMan) v.5.4.1 for Windows (The Nordic Cochrane Center, Copenhagen, Denmark). Differences between the intervention and control groups were assessed. In the analysis of clinical efficacy, dichotomous data were assessed in terms of risk ratios (RRs), and continuous data were assessed in terms of mean differences (MDs). Dichotomous and continuous variables were expressed as efficacy values with 95% confidence intervals (CIs). In cases of outcome variables assessed using different scales, the standardized MD (SMD) was used instead of the weighted MD (WMD). If heterogeneity was detected (defined by heterogeneity tests with a chi-square test of *p* < 0.1 and Higgins of *I*^2^ ≥ 50%), subgroup analyses were performed to determine the cause of clinical heterogeneity. A random-effects model was used to assess combined effect sizes from efficacy variables, and substantial clinical heterogeneity was expected across the included studies based on diversity among the interventions, study designs, and other conditions. An albatross plot showing the effects of direction and size range by *p*-value and the given sample size was generated for each included study. Publication bias was assessed using funnel plots if more than 10 studies were available (15).

## Results

A total of 1,110 studies were identified in the search of 12 databases. A total of four studies were added based on other identified sources. A total of 344 articles were eliminated due to duplication. A total of 770 studies were screened by reading the titles and abstracts, and 63 articles remained. Notably, 33 articles were excluded due to the reasons described in Figure 1 after the full texts of the 63 articles were read. Finally, 30 studies (16–45) met the inclusion criteria, and a meta-analysis was conducted. The PRISMA flowchart of the search process is shown in Figure 1.

### Characteristics of included studies

A total of 25 studies were conducted in China (19–28, 30–42, 44, 45), two in the United Kingdom (29, 43), one in the United States (16), one in Turkey (18), and one in Hong Kong (17). The main characteristics of the 30 included studies are presented in Table 1. Seven studies compared AT with sham AT (16–22), and 13 studies compared AT with drug therapy (18, 20, 23–33). Two of the studies had two control groups: sham AT and drug therapy (18, 20). One study had two intervention groups: AT and AT plus drug therapy (25). Ten studies used AT plus drug therapy as an intervention and drug therapy alone as a control (25, 34–42). Three studies compared AT plus standard care with standard care alone (43–45).

**Table 1:** Summary of the characteristics of the included studies.

**References**	**Sample size (randomized/analyzed)** **Sex (M/F)** **OAB duration (years)** **Age (years)**	**Intervention group (regimen)**	**Comparison group (regimen)**	**Outcome measures**	**Main results**	**Authors conclusion**	**Adverse effects**
Emmons and Otto (16)	85/74 (0/85) n.r. A: 53; B: 51	(A) MA (20 min, once weekly for 4 weeks, *n* = 38)	(B) Sham MA (penetrating, not related acupuncture points, 20 min, once weekly for 4 weeks, *n* = 36)	1) Frequency^†^ 2) Incontinence	1) SMD −0.55 (−1.02, −0.09), *p* < 0.05 2) SMD −0.57 (−1.04, −0.11), *p* < 0.05	“… MA … significant improvements...”	Bleeding or bruising (n.r.: 23%), discomfort with needle (n.r.: 25%)
Lin et al. (17)	100/96 (45/55) n.r. A: 69.0; B: 67.9	(A) MA (30 min, twice weekly for 8 weeks, *n* = 49)	(B) Sham MA (non-penetrating, same acupuncture points, 30 min, twice weekly for 8 weeks, *n* = 48)	1) OABSS^†^ 2) Frequency^†^ 3) Incontinence^†^	1) MD −0.20 (−1.24, 0.84), NS 2) SMD −0.11 (−0.52, 0.29), NS 3) SMD 0.26 (−0.14, 0.66), NS	“… beneficial effect of MA…”	None
Aydogmus et al. (18)	90/82 (0/90) n.r. 38	(A) MA (20 min, twice weekly for 4 weeks, *n* = 28)	(B) Sham MA (non-penetrating, same acupuncture points, 20 min, twice weekly for 4 weeks, *n* = 24) (C) Drug therapy (Solifenacin, oral, 5 mg, once daily for 4 weeks, *n* = 30)	1) OABSS^†^ 2) AEs^†^	1) A vs. B: *P* < 0.001, A vs. C: NS 2) A vs. C: RR 0.03 (0.00, 0.43), *P* < 0.0001	“…MA may be considered another treatment option.”	Dry mouth (C: 19)
Tang et al. (19)	64/64 (0/64) A: 3.2; B: 3.2 A: 41.88; B: 46.19	(A) EA (20 min, 3 times weekly for 1week, *n* = 33)	(B) Sham EA (5 mm penetrating, same acupuncture points, current, 20 min, 3 times weekly for 1week, *n* = 31)	1) OABSS^†^ 2) Response rate	1) MD −1.16 (−1.38, −0.94), *p* < 0.00001 2) RR 3.76 (1.78, 7.94), *p* < 0.00001	“EA can effectively improve … with OAB”	n.r.
Yang (20)	37/33 (10/27) n.r. A: 60.5; B: 62.8; C: 49.4	(A) EA (30 min, 3 times weekly for 8 weeks, *n* = 12)	(B) Sham EA (non-penetrating, same acupuncture points, 30 min, 3 times weekly for 8 weeks (*n* = 13) (C) Drug therapy (Solifenacin, oral, 5 mg, once daily for 8 weeks, *n* = 8)	1) Frequency^†^ 2) Incontinence^†^ 3) AEs	1) A vs. B: SMD −0.48 (−1.28, 0.32), NS, A vs. C: MD 2.20 (−0.45, 4.85), NS 2) A vs. B: SMD −0.37 (−1.16, 0.42), NS A vs. C: MD −0.33 (−2.75, 2.09), NS 3) A vs. C: RR not estimable	“EA …may be a good way to solve OAB”	None
Zhang et al. (21)	50/45 (0/50) A: 2.1; B: 1.9 A: 39.0; B: 43.5	(A) EA (30 min, 5 times weekly for 6 weeks, *n* = 23)	(B) Sham EA (superficial penetrating, non-acupuncture points, no-current, 30 min, 5 times weekly for 6 weeks, *n* = 22)	OABSS^†^	MD −2.40 (−3.88, −0.92), *p* < 0.01	“EA … effective, safe …”	Pain at needling sites (A: 3, B: 2)
Yu (22)	22/22 (7/15) A: 3.0; B: 5.0 A: 68.0; B: 62.6	(A) EA (30 min, 3 times weekly for 4 weeks, *n* = 13)	(B) Sham EA (penetrating, acupuncture points, no current, 30 min, 3 times weekly for 4 weeks, *n* = 9)	1) Frequency^†^ 2) Incontinence^†^	1) SMD −0.61 (−1.49, 0.26), NS 2) SMD 0.00 (−0.85, 0.85), NS	“EA… has specific effect, … safe…”	Thumb-sized crape myrtle (n.r.: 1)
Wang et al. (23)	60/60 (0/60) ≥0.5 35–60	(A) MA (30 min, once daily for 4 weeks, *n* = 30)	(B) Drug therapy (solifenacin, oral, 5 mg, once daily for 4 weeks, *n* = 30)	1) OABSS^†^ 2) Frequency^†^ 3) Response rate	1) MD −0.20 (−1.14, 0.74), NS 2) MD −0.40 (−2.76, 1.96), NS 3) RR 1.04 (0.86, 1.25), NS	“MA can safely and effectively improve …”	n.r.
Wang and Lin (24)	60/60 (52/8) n.r. 61	(A) MA (30 min, 6 times weekly for 4 weeks, *n* = 30)	(B) Drug therapy (solifenacin, oral, 5 mg, once daily for 4 weeks, *n* = 30)	1) Frequency^†^ 2) Incontinence^†^ 3) Response rate	1) MD 0.80 (−0.27, 1.87), NS 2) MD 0.00 (−0.35, 0.35), NS 3) RR 0.93 (0.76, 1.13), NS	“MA … improve …”	n.r.
Hou et al. (25)	90/90 (26/64) 0.6 51.5	(A) MA (30 min, once daily for 10 days and rest 3 days, total 36 days, *n* = 30) (B) MA(30 min, once daily for 10 days and rest 3 days, total 36 days, *n* = 30), plus C	(C) Drug therapy (solifenacin, oral, 5 mg, once daily for 36 days, *n* = 30)	1) OABSS^†^ 2) Frequency^†^ 3) Response rate	1) A vs. C: MD 1.07 (−0.05, 2.19), NS, B vs. C: MD −2.21 (−3.64, −0.78), *p* < 0.01 2) A vs. C: MD 1.87 (1.25, 2.49), *p* < 0.00001, B vs. C: MD −1.68 (−2.60, −0.76), *p* < 0.001 3) A vs. C: RR 0.95 (0.67, 1.34), NS, B vs. C: RR 1.24 (0.94, 1.63), NS	“…combined use of acupuncture and M receptor antagonists is significantly better …”	n.r.
Yuan et al. (26)	272/240 (0/272) n.r. A: 57.5; B: 58.2	(A) MA (20 min, once weekly for 4 weeks, *n* = 118)	(B) Drug therapy (tolterodine, oral, 2 mg, twice daily for 4 weeks, *n* = 122)	1) Frequency^†^ 2) Incontinence^†^ 3) AEs^†^	1) MD 0.10 (−0.51, 0.71), NS 2) MD 0.20 (−0.01, 0.41), NS 3) RR 0.85 (0.36, 1.97), NS	“...MA is safe with significant improvements...”.	Needling pain (A: 9), dry mouth (B: 11)
Wang and Shi (27)	40/40 (0/40) n.r. 51–79	(A) MA (20 min, once daily for 4 weeks, *n* = 20)	(B) Drug therapy (tolterodine oral, 2 mg, twice daily for 4 weeks, *n* = 20)	1) Frequency^†^ 2) Response rate 3) AEs^†^	1) MD −1.20 (−4.24, 1.84), NS 2) RR 1.29 (0.93, 1.77), NS 3) RR 0.20 (0.01, 3.92), NS	“...the total effective rate in the treatment group was better …”	Dry mouth (B: 2)
Yu and Wang (28)	44/44 (22/22) A: 0.5–5; B: 0.3–4 A: 35.2; B: 37.1	(A) MA (n.r. twice daily for 2 weeks, *n* = 24)	(B) Drug therapy (tolterodine oral, 2 mg, twice daily for 2 weeks, *n* = 20)	Response rate	RR 1.52 (0.98, 2.34), *p* < 0.05	“MA.. is better…”	n.r.
Kelleher et al. (29)	39/36 (0/39) A: 5.2; B: 4.9 A: 51.2; B: 48.1	(A) MA (10 min, once weekly for 6 weeks, *n* = 20)	(B) Drug therapy (oxybutynin, oral, 5 mg, twice daily for 6 weeks, *n* = 16)	1) Frequency^†^ 2) AEs^†^	1) NS 2) RR 0.27 (0.13, 0.56), *P* < 0.0001	“AT is … effective.. with few adverse effect…”	Discomfort (A: 2), headache (A: 3), dry mouth (B: 21), headaches, dizziness, GI upset, transient visual impairment (B: >10), unacceptable side effect (n.r. in detail, B: 3)
Zhu et al. (30)	60/57 (30/30) A: 1.6; B:1.7 A: 51.6; B: 49.4	(A) EA (45 min, 3 times weekly for 28 days, *n* = 28)	(B) Drug therapy (solifenacin, oral, 5 mg, once daily for 28 days, *n* = 29)	1) OABSS^†^ 2) Frequency^†^	1) MD 0.50 (0.23, 0.77), *p* < 0.001 2) MD 0.60 (−0.03, 1.23), NS	EA is effective and safe	Dry mouth (B: 1), constipation (B: 1)
Zhu and Bi (31)	90/90 (32/58) A: 4.3; B: 4.6 A: 63.4; B: 63.6	(A) EA (30 min, once daily for 2 weeks, *n* = 45)	(B) Drug therapy (tolterodine, oral, 2 mg, twice daily) or (solifenacin, oral, 5 mg, once daily) for 2 weeks, *n* = 45)	Response rate	RR 1.42 (1.03, 1.95), *p* < 0.05	“EA …can significantly improve …”	n.r.
Zhang et al. (32)	104/97 (62/44) A: 2.4; B: 2.6 A: 66.5; B: 68.2	(A) EA (20 min, 6 times weekly for 2 weeks, *n* = 48)	(B) Drug therapy (tolterodine, oral, 4 mg, once daily for 3 weeks, *n* = 49)	1) OABSS^†^ 2) Response rate 3) AEs	1) MD −3.00 (−4.00, −2.00), *p* < 0.00001 2) RR 1.07 (0.94, 1.21), NS 3) RR 0.68 (0.20, 2.26), NS	“EA … can improve … better than the tolterodine tartrate…”	10 minor adverse (A: 4); (B: 6)
Chen et al. (33)	48/48 (48/0) 1–32 51	(A) EA (30 min, once daily for 14 days, *n* = 24)	(B) Drug therapy (tolterodine, oral, 2 mg, twice daily for 14 days, *n* = 24)	Response rate	RR 1.11 (0.86, 1.43), NS	“EA is an effective…”	n.r.
Su et al. (34)	67/67 (25/42) A: 2.4; B: 2.2 A: 50.3; B: 51.6	(A) MA (20 min, 3 times weekly for 12 weeks, *n* = 34), plus B	(B) Drug therapy (tolterodine, oral, 4 mg, once daily for 12 weeks, *n* = 33)	1) OABSS^†^ 2) Response rate	1) MD −1.94 (−2.59, −1.29), *p* < 0.00001 2) RR 1.14 (0.98, 1.34), NS	“…MA with tolterodine… can significantly improve...”	Dry mouth (A: 3, B: 5), constipation (A: 2, B: 4), dry eyes (A: 3, B: 2)
Mao (35)	60/58 (26/64) 0.4–1.7 51.5	(A) MA (30 min, once daily for 10 days and rest 3 days, total 36 days, *n* = 30), plus B	(B) Drug therapy (solina, oral, 5 mg, once daily for 36 days, *n* = 28)	1) OABSS^†^ 2) Response rate	1) MD −2.21 (−3.65, −0.77), *p* < 0.01 2) RR 1.28 (0.95, 1.71), NS	“MA…is safe and effective…”	None
Li et al. (36)	60/60 (60/0) A: 11.8; B: 10.5 A: 61.5; B: 60.9	(A) MA (40 min, manipulate at 20 min, once daily for 12 weeks, *n* = 30), plus B	(B) Drug therapy (Finasteride, oral, 5 mg, twice daily for 12 weeks, *n* = 30)	Response rate	RR 1.29 (0.99, 1.67), *p* < 0.05	“MA …significantly improve the symptoms....”	n.r.
Xiong et al. (37)	40/40 (0/40) A: 4.1; B: 4.2 A: 45.8; B: 46.2	(A) EA (30 min, once per 2 days for 12 weeks, *n* = 20), plus B	(B) Drug therapy (SOLIFENACIN, oral, 5 mg, once daily daily for 12 weeks, *n* = 20)	1) OABSS^†^ 2) Frequency^†^	1) MD −3.76 (−4.52, −3.00), *p* < 0.00001 2) MD −3.62 (−5.61, −1.63), *p* < 0.01	“…EA… significantly improve …”	n.r.
Chen et al. (38)	74/74 (0/74) A: 1.4; B: 1.3 A: 58; B: 60	(A) EA (30 min, 5 times weekly for 4 weeks, *n* = 37), plus B	(B) Drug therapy (Solifenacin, oral, 5 mg, once daily daily for 4 weeks, *n* = 37)	1) OABSS^†^ 2) Frequency^†^ 3) Incontinence^†^ 4) Response rate	1) MD −1.30 (−1.92, −0.68), P < 0.001 2) MD −1.93 (−2.27, −1.59), *p* < 0.00001 3) MD −0.44 (−0.59, −0.29), p < 0.00001 4) RR 1.21 (1.00, 1.45), *p* < 0.05	“EA plus Solifenacin … effective and safe … OAB”	n.r.
Zhao (39)	68/68 (30/38) A: 1.2; B: 1.3 A: 33.9; B: 35.0	(A) EA (20 min, once daily for 30days, *n* = 34), plus B	(B) Drug therapy (solifenacin, oral, 5 mg, once daily daily for 30 days, *n* = 34)	Frequency^†^	MD −3.35 (−4.96, −1.74), *p* < 0.001	“EA combined with solifenacin … improve…”	Dry mouth (B: 1)
Chen et al. (40)	96/96 (0/96) 3.8 44	(A) EA (30 min, once daily for 14 days, *n* = 48), plus B	(B) Drug therapy (tolterodine, oral, 2 mg, twice daily for 14 days, *n* = 48)	1) Frequency^†^ 2) Incontinence^†^ 3) Response rate	1) MD −2.50 (−3.18, −1.82), *p* < 0.00001 2) MD −0.60 (−0.97, −0.23), p < 0.01 3) RR 1.26 (1.04, 1.52), *p* < 0.05	“EA combined with tolterodine… is better…”	None
Wang et al. (41)	120/120 (n.r.) n.r. n.r	(A) EA (30 min, once daily for 3 months, *n* = 60), plus B	(B) Drug therapy (tolterodine, oral, 2 mg, twice daily for 3 months, *n* = 60)	Frequency^†^	MD −0.13 (−0.75, 0.49), NS	“EA... have significant clinical effect”	n.r.
Liao et al. (42)	67/67 (27/40) A: 2.7; B: 2.5 A: n.r.; B: 43	(A) EA (30 min, once daily for 4 weeks, *n* = 35), plus B	(B) Drug therapy (tolterodine, oral, 2 mg, twice daily for 4 weeks, *n* = 32)	Frequency^†^	MD −8.00 (−11.62, −4.38), *p* < 0.00001	“EA … can significantly improve…”	n.r.
Hargreaves et al. (43)	30/29 (0/30) Not clearly reported A: 57.2; B: 54.5	(A) MA (30 min, 6 sessions for 8 weeks, *n* = 16), plus B	(B) Usual care (fluid intake, caffeine modification, bladder health advice, pelvic floor exercises, weight reduction, smoking cessation advice, 8 weeks, *n* = 13)	1) Frequency^†^ 2) Incontinence^†^	1) MD 0.12 (−1.73, 1.97), NS 2) MD 0.88 (−1.09, 2.85), NS	“may be benefits … MA”	11 minor adverse Bleeding (A: 4); bruising (A: 6); vomiting (A: 1)
Xie and Yang (44)	71/71 (0/71) n.r. A: 45.9; B: 46.3	(A) MA (30 min, once daily for 2 months, *n* = 36), plus B	(B) Usual care (urination training, bladder training, pelvic floor muscle exercise, health education, 2 months, *n* = 35)	1) Frequency^†^ 2) Incontinence^†^ 3) Response rate	1) MD −2.10 (−2.82, −1.38), *p* < 0.00001 2) MD −0.40 (−0.68, −0.12), *p* < 0.01 3) RR 1.30 (1.01, 1.67), *p* < 0.05	“MA combined with behavioral intervention… effective..”	n.r.
Li et al. (45)	60/60 (0/60) 3.5 36.3	(A) MA (10 min, 6 times weekly for 5 weeks, *n* = 30), plus B	(B) Usual care (bladder training, biofeedback therapy, pelvic floor muscle training, 5 weeks, *n* = 30)	1) Frequency^†^ 2) Incontinence^†^	1) RR 0.50 (0.22, 1.16), NS 2) RR 0.44 (0.10, 1.97), NS	“MA and nursing intervention … effectively improve…”	n.r.

MA, manual acupuncture; EA, electroacupuncture; n.r., not reported; MD, mean difference; SD, standard deviation; OABSS, over active bladder symptom score; RR, response rate.

^†^A lower score indicates a better condition.

A higher score indicates a better condition.

Rationales for using AT were reported in 97% of studies. These studies were mainly based on traditional Chinese medicine (TCM) theory (16–29, 31, 33–36, 38–45).

All studies reported acupoints, and the more frequently used acupoints included SP6 (17 studies) (16–19, 24–29, 31, 34, 35, 39, 41, 43, 44), BL23 (17 studies) (17, 19, 23–25, 27–29, 31, 35, 38–42, 44, 45), BL28 (15 studies) (16, 17, 23–25, 27–29, 31, 35, 38, 39, 42, 44, 45), CV4 (15 studies) (16–18, 23, 25–27, 29, 31, 34, 35, 39, 40, 43, 44), BL32 (15 studies) (17, 19, 21, 23–25, 27, 31–33, 35, 39, 41, 44, 45), and CV3 (11 studies) (19, 23, 25, 27, 29, 31, 34, 35, 39, 43, 44).

In total, 19 studies selected the “de qi” response (16, 18, 21, 22, 26, 31, 33–36, 38–40, 42, 43). All studies reported needle stimulation methods. The most frequently used retention time was 30 min (17 studies) (17, 21–24, 31, 33, 35, 37, 38, 40–44). The treatment period varied from 1 to 12 weeks, and the largest number of studies was conducted over 4 weeks (16, 18, 22–24, 26, 27, 30, 38, 42). The details of the STRICTA domains are shown in Supplementary material 2.

### Risk of bias in the included studies

**Figure 2:**
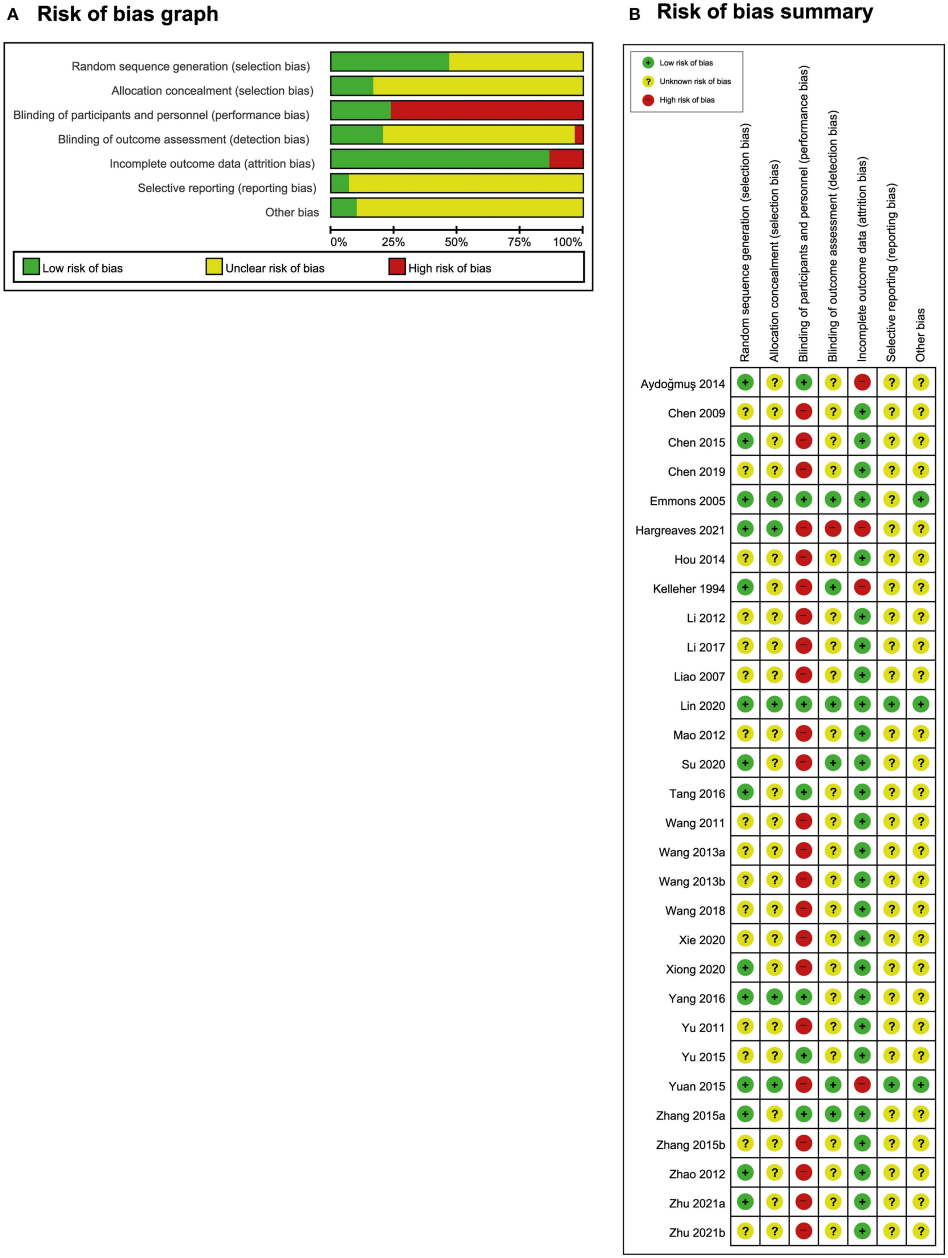
Risk of bias. **(A)** Risk-of-bias graph and **(B)** risk-of-bias summary: The present authors' judgments regarding the risk of each form of bias in all included studies.

The risk of bias in these studies is presented in Figure 2. A total of 14 studies had a low risk of bias regarding random sequence generation (16–21, 26, 29, 30, 34, 37, 39, 40, 43), whereas 16 studies did not provide detailed information about their random generation method (22–25, 27, 28, 31–33, 35, 36, 38, 41, 42, 44, 45). Notably, five studies reported the allocation concealment method (16, 17, 20, 26, 43), whereas the other 25 studies did not mention the allocation concealment method (18, 19, 21–25, 27–42, 44, 45). A total of seven studies had a low risk of performance bias given that these studies selected AT as the intervention and sham AT as the control (16–22). The remaining 23 studies had a high risk of performance bias. Given the distinct differences between the intervention and control groups, patients or clinicians could not be blinded (18, 23–45). In five studies, blinding was performed by the investigators (16, 17, 21, 26, 29). In one study, blinding was not performed, but it was judged to not affect the evaluation of results given that every participant wrote their symptom scores by themselves (34). In one study, the practitioner and outcome investigators were the same people given the lack of resources (43). The other 23 studies did not report the blinding of the outcome assessor (18–20, 22–25, 27, 28, 30–33, 35–42, 44, 45). Twenty-six studies had a low risk of attrition bias given that 19 of these studies had no dropout rate (19, 22–25, 27, 28, 31, 33, 34, 36–42, 44, 45); six had a low dropout rate, which is small enough to not affect the results (17, 20, 21, 30, 32, 35); and one had moderate dropout, but the reasons were similar in both the intervention and control groups (16). Four studies had high dropout rates (18, 26, 29, 43). Two studies were conducted in accordance with their protocols (17, 26), whereas the other 28 studies did not report the protocol or disclose all prespecified and expected results (16, 18–25, 27–45). Three studies reported adequate details to eliminate various biases (16, 17, 26), whereas the other 27 studies did not report sufficient information (18–25, 27–45).

### Effects of interventions

#### AT vs. sham AT

##### OABSS

**Figure 3:**
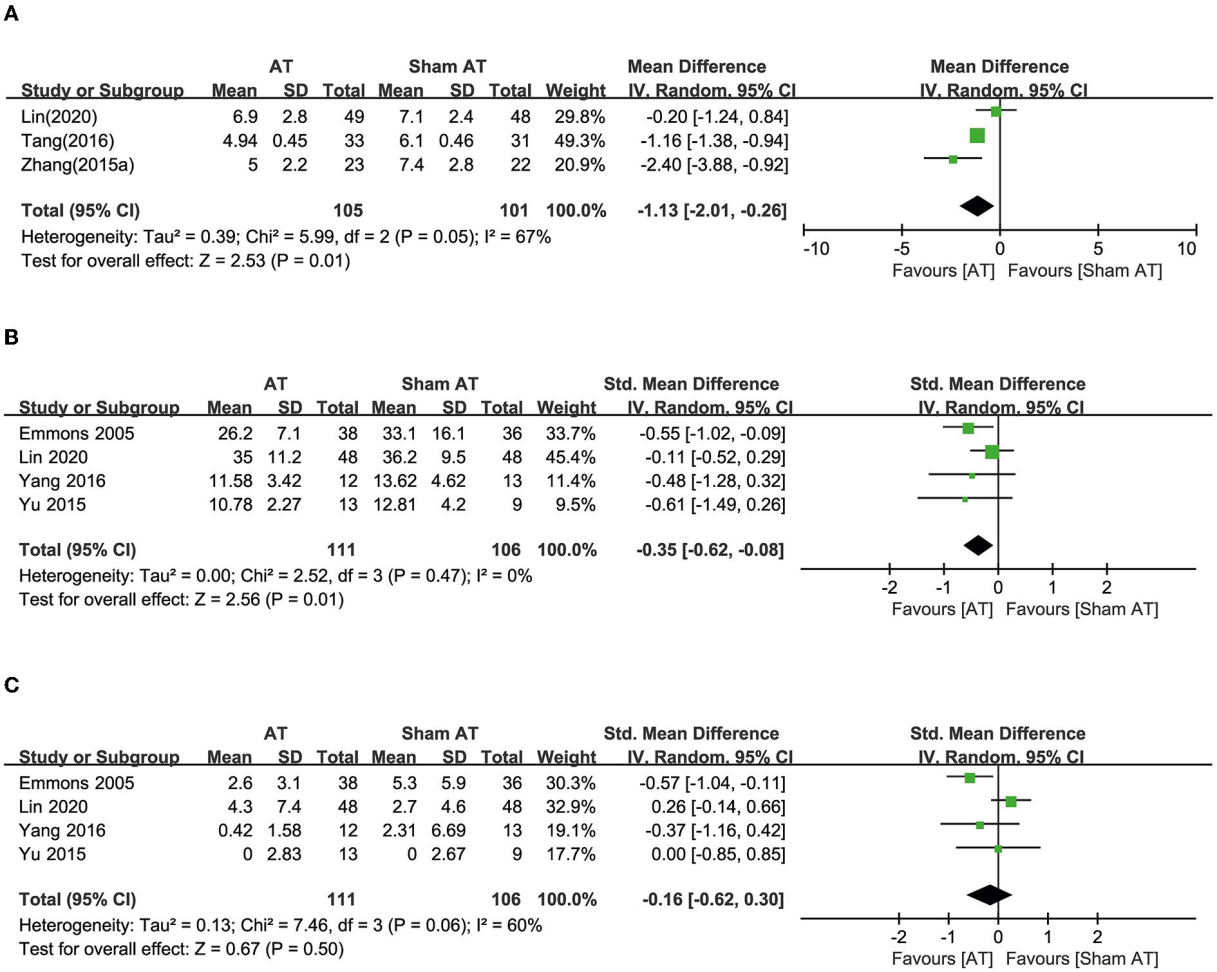
Forest plot of **(A)** OABSS, **(B)** frequency, and **(C)** incontinence according to the comparison of AT vs. sham AT.

Seven RCTs compared the effects of AT with sham AT (16–22). Three RCTs reported OABSS. In one study, AT exhibited an effect equivalent to sham AT (17), whereas the other two studies showed favorable effects of AT on reducing OABSS compared with sham AT (19, 21). Meta-analysis revealed that AT showed a more favorable effect than sham AT, and heterogeneity was high (MD: −1.13, 95% CI: −2.01 to −0.26, *p* = 0.01, *I*^2^ = 67, Figure 3A).

##### Frequency

Four RCTs reported urinary frequency, and one of these studies showed a favorable effect of AT compared with sham AT (16). The other three RCTs showed equivalent effects (17, 20, 22). Meta-analysis revealed that AT had more favorable effects than sham AT on reducing urinary frequency (SMD: −0.35, 95% CI: −0.62 to −0.08, *p* = 0.01, *I*^2^ = 0%, Figure 3B).

##### Incontinence

Four RCTs reported urinary incontinence. One showed a favorable effect of AT compared with sham AT (16), but three showed equivalent effects (17, 20, 22). Meta-analysis revealed that AT exhibited effects equivalent to sham AT on reducing urinary incontinence (SMD: −0.16, 95% CI: −0.62 to 0.30, *p* = 0.50, *I*^2^ = 60%, Figure 3C).

##### Response rate

One RCT reported the response rate and showed a favorable effect of AT compared with sham AT (RR: 3.76, 95% CI: 1.78 to 7.94, *p* = 0.0005) (19).

#### AT vs. drug therapy

##### OABSS

**Figure 4:**
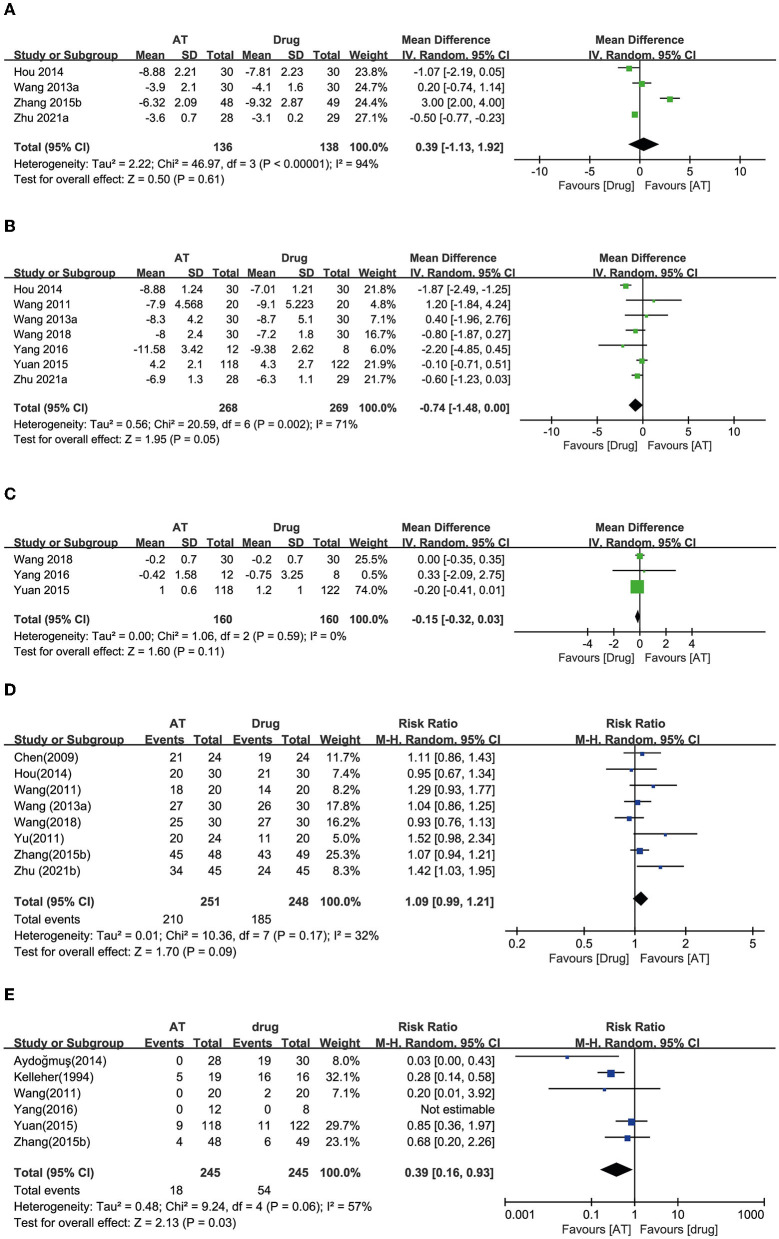
Forest plot of **(A)** OABSS, **(B)** frequency, **(C)** incontinence, **(D)** response rate, and **(E)** adverse effects according to the comparison of AT vs. drug therapy.

Thirteen RCTs used anticholinergic conventional drug therapy as a control (18, 20, 23–33). Four RCTs reported OABSS. Two showed equivalent effects of AT (23, 25), and one showed a favorable effect of AT (32). In contrast, the other study reported a favorable effect of drug therapy (30). A meta-analysis demonstrated equivalent effects of AT with drug therapy on reducing OABSS (MD: −0.39, 95% CI: −1.92 to 1.13, *p* = 0.61, *I*^2^ = 94%, Figure 4A).

##### Frequency

Seven RCTs reported urinary frequency, and six of these studies showed equivalent effects of AT with drug therapy on reducing urinary frequency (20, 23, 24, 26, 27, 30). The other reported a favorable effect of drug therapy (25). Meta-analysis revealed that AT exhibited effects equivalent to drug therapy on reducing urinary frequency (MD: 0.74, 95% CI: −0.00 to 1.48, *p* = 0.05, *I*^2^ = 71%, Figure 4B).

##### Incontinence

Three RCTs reported the number of daily incontinence episodes, and all of them showed that AT had equivalent effects to drug therapy (20, 24, 26). Through the meta-analysis, AT had equivalent effects with drug therapy on reducing urinary incontinence (MD: 0.15, 95% CI: −0.03 to 0.32, *p* = 0.11, *I*^2^ = 0%, Figure 4C).

##### Response rate

Eight RCTs reported the response rate, and seven of them showed equivalent effects of AT and drug therapy (23–25, 27, 28, 32, 33). One RCT showed a favorable effect of AT compared with drug therapy (31). Through the meta-analysis, AT showed equivalent effects as drug therapy on the response rate (RR: 1.09, 95% CI: 0.99 to 1.21, *p* = 0.09, *I*^2^ = 32%, Figure 4D).

##### AEs

Six studies reported the AEs of participants, and three of the studies showed an incidence of AT equivalent to that of drug therapy (26, 27, 32). Two RCTs reported a significantly lower incidence of side effects with AT compared with drug therapy (18, 29). AEs could not be estimated in one RCT because it reported that both groups experienced no AEs (20). Meta-analysis revealed that AT showed a significantly lower incidence of AEs compared with drug therapy (RR: 0.39, 95% CI: 0.16 to 0.93, *p* = 0.03, *I*^2^ = 57%, Figure 4E).

#### AT plus drug therapy vs. drug therapy

##### OABSS

**Figure 5:**
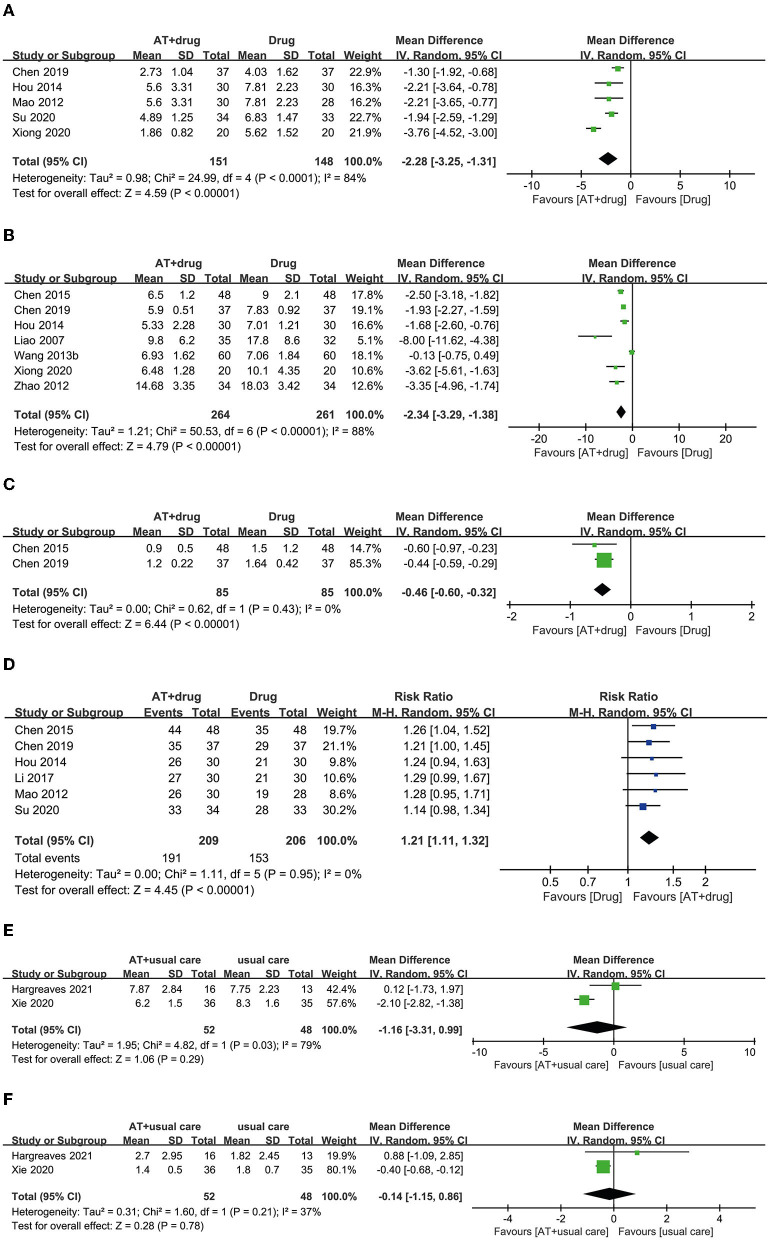
Forest plot of **(A)** OABSS, **(B)** frequency, **(C)** incontinence, and **(D)** response rate according to the comparison of AT plus drug therapy vs. drug therapy. **(E)** Frequency, **(F)** incontinence according to the comparison of AT plus usual care vs. usual care.

A total of 10 RCTs used the combination of AT and drug therapy as an intervention, and the same anticholinergic conventional drug therapies served as a control (25, 34–42). Five RCTs reported OABSS, and all of them showed favorable effects of AT plus drug therapy for reducing OABSS compared with drug therapy alone (25, 34, 35, 37, 38). The meta-analysis revealed that the combination of AT and drug therapy showed favorable effects compared with drug therapy alone (MD: −2.28, 95% CI: −3.25 to −1.31, *p* < 0.00001, *I*^2^ = 84%, Figure 5A).

##### Frequency

Seven RCTs reported urinary frequency, and six of them showed favorable effects of AT plus drug therapy for reducing urinary frequency compared with drug therapy alone (25, 37–40, 42). In one RCT, the combination of AT and drug therapy had an equivalent effect on drug therapy alone (41). Meta-analysis revealed that AT plus drug therapy had favorable effects on reducing urinary frequency compared with drug therapy alone (MD: −2.34, 95% CI: −3.29 to −1.38, *p* < 0.00001, *I*^2^ = 88%, Figure 5B).

##### Incontinence

Two RCTs reported the number of patients experiencing urinary incontinence, and both RCTs showed more favorable effects of AT plus drug therapy for reducing urinary incontinence than drug therapy alone (38, 40). A meta-analysis also revealed favorable effects of the combination of AT and drug therapy over drug therapy alone (MD: −0.46, 95% CI: −0.60 to −0.32, *p* < 0.00001, *I*^2^ = 0%, Figure 5C).

##### Response rate

Six RCTs reported the response rate, and four RCTs showed equivalent effects for the combination of AT plus drug therapy and drug therapy alone (25, 34–36). However, two RCTs showed that AT combined with drug therapy had more favorable effects on the response rate than drug therapy alone (38, 40). Meta-analysis revealed that the combination of AT and drug therapy had a more favorable effect on the response rate than drug therapy alone (RR: 1.21, 95% CI: 1.11 to 1.33, *p* < 0.00001, *I*^2^ = 0%, Figure 5D).

#### AT plus usual care vs. usual care

##### Frequency

Three studies selected AT plus usual care as an intervention and usual care as a control (43–45). Two RCTs reported urinary frequency. One of these RCTs showed equivalent effects (43). In contrast, the other showed a more favorable effect of AT plus usual care compared with usual care alone (44). Meta-analysis revealed equivalent effects of AT plus usual care and usual care alone for reducing urinary frequency (MD: −1.16, 95% CI: −3.31 to 0.99, *p* = 0.29, *I*^2^ = 79%, Figure 5E).

##### Incontinence

Two RCTs reported urinary incontinence. One RCT showed an equivalent effect (43), whereas the other showed a more favorable effect of AT plus usual care than usual care alone (44). The meta-analysis revealed equivalent effects of AT plus usual care and usual care alone for reducing urinary incontinence (MD: −0.14, 95% CI: −1.15 to 0.86, *p* = 0.78, *I*^2^ = 37%, Figure 5F).

##### Response rate

Only one RCT reported the response rate, and a more favorable effect of AT plus usual care was noted compared with usual care alone (44).

#### Total AEs

A total of 15 studies did not report AEs (19, 23–25, 28, 30, 31, 33, 36–38, 41, 42, 44, 45). In seven studies, AEs of AT did not occur (18, 20, 27, 34, 35, 39, 40). Eight studies reported minor AEs, such as needling pain, bruising, and bleeding. However, no severe AEs were reported (16, 17, 21, 22, 26, 29, 32, 43).

#### Albatross plot and publication bias

**Figure 6:**
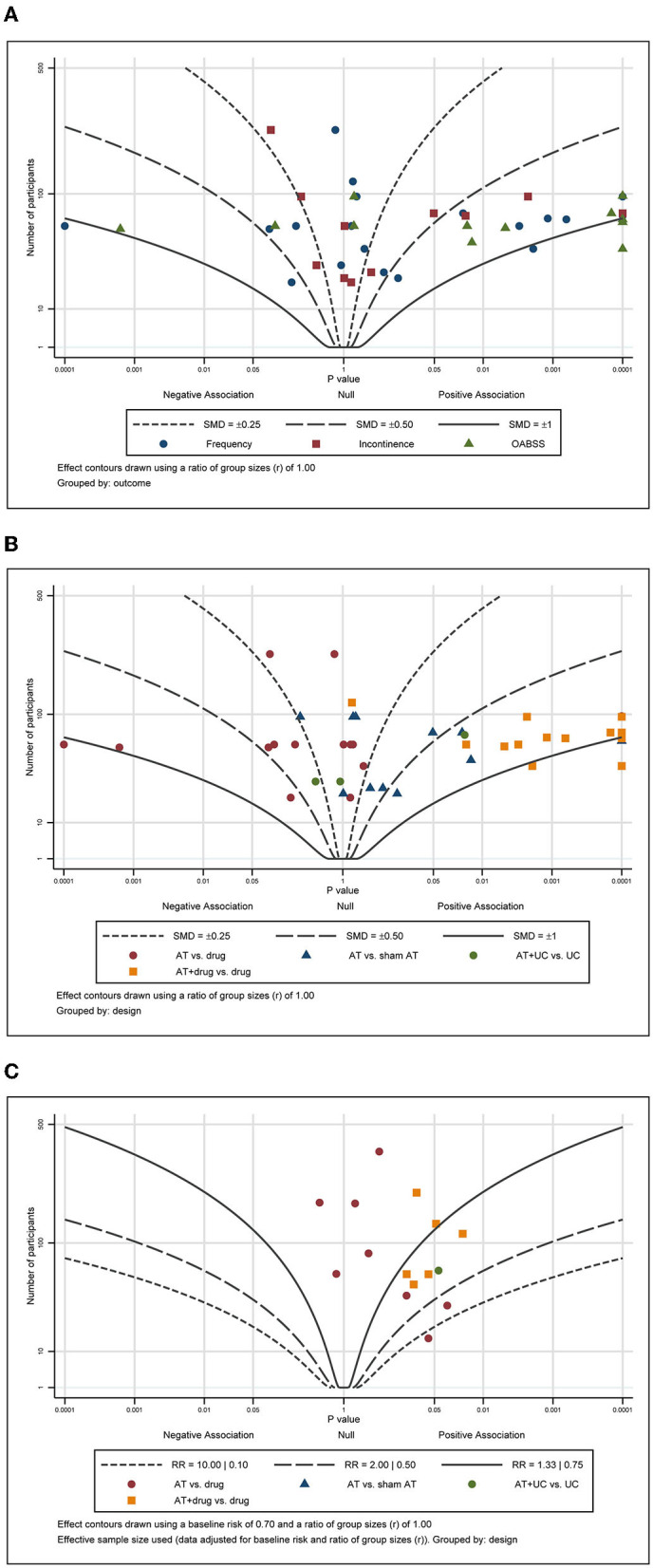
Albatross plot of **(A)** frequency, **(B)** incontinence, and **(C)** response rate according to the comparison of MA plus standard care vs. standard care.

For the continuous outcomes, including pain, function, and QoL, most points were scattered and accumulated on the right side of the plot with many points clustered around the null line, failing to show specific effects of AT on these outcomes (Figures 6A, B). For the total effective rate, the points were scattered across the contour lines (Figure 6C). All the points were clustered on the positive association side of the plot, indicating that ginseng is favorable for the management of OAB by AT.

#### Summary of findings

The certainty of evidence (CoE) was assessed using the GRADEpro program, and a summary of the findings, including studies with low or very low CoE, is shown in Table 2.

**Table 2:** Summary of findings.

**Outcome**	**No of Participants (studies)**	**Certainty of evidence (GRADE)**	**Relative effect (95% CI)**	**Anticipated absolute effects** ^*^ **(95% CI)**	
				**Risk with sham AT**	**Risk with AT (MA** + **EA)**
**AT compared to sham AT for overactive bladder**					
OABSS	205 (3 RCTs)	⊕○○○ Very low^a^, ^b^, ^c^, ^d^		The mean OABSS was −1.13	MD 1.13 lower (2.01 lower to 0.26 lower)
Frequency	217 (4 RCTs)	⊕⊕○○ Low^a^, ^d^		The mean frequency was −0.35	SMD 0.35 lower (0.62 lower to 0.08 lower)
Incontinence	217 (4 RCTs)	⊕○○○ Very low^a^, ^b^, ^d^		The mean incontinence was −0.16	SMD 0.16 lower (0.62 lower to 0.3 higher)
Response rate	64 (1 RCT)	⊕⊕○○ Very low^a^, ^d^	RR 3.76 (1.78–7.94)	194 per 1,000	534 more per 1,000 (151 more to 1,000 more)
**AT compared to drug therapy for overactive bladder**					
OABSS	274 (4 RCTs)	⊕○○○ Very low^b^, ^d^, ^e^	–	The mean OABSS was −0.39	MD 0.39 lower (1.92 lower to 1.13 higher)
Frequency	537 (7 RCTs)	⊕○○○ Very low^a^, ^b^, ^c^	–	The mean frequency was 0.74	MD 0.74 higher (0 higher to 1.48 higher)
Incontinence	320 (3 RCTs)	⊕○○○ Very low^a^, ^b^, ^d^	–	The mean incontinence was 0.15	MD 0.15 higher (0.03 lower to 0.32 higher)
Response rate	499 (8 RCTs)	⊕○○○ Low^b^, ^e^	RR 1.09 (0.99–1.21)	746 per 1,000	67 more per 1,000 (7 fewer to 157 more)
Adverse effects	491 (6 RCTs)	⊕○○○ Low^a^, ^b^, ^c^	RR 0.39 (0.16–0.93)	220 per 1,000	134 fewer per 1,000 (185 fewer to 15 fewer)
**AT** + **drug compared to drug for overactive bladder**					
OABSS	239 (4 RCTs)	⊕○○○ Very low^a^, ^b^, ^d^	–	The mean OABSS was −2.28	MD 2.28 lower (3.25 lower to 1.31 lower)
Frequency	465 (6 RCTs)	⊕⊕○○ Low^a^, ^d^	–	The mean frequency was −2.34	MD 2.34 lower (3.29 lower to 1.38 lower)
Incontinence	170 (2 RCTs)	⊕⊕○○ Low^a^, ^d^	–	The mean incontinence was −0.46	MD 0.46 lower (0.6 lower to 0.32 lower)
Response rate	355 (5 RCTs)	⊕⊕○○ Low^a^, ^d^	RR 1.21 (1.11 to 1.32)	743 per 1,000	156 more per 1,000 (82 more to 238 more)
**AT** + **usual care compared to usual care for overactive bladder**					
Frequency	100 (2 RCTs)	⊕○○○ Very low^a^, ^b^, ^d^	–	The mean frequency was −1.16	MD 1.16 lower (3.31 lower to 0.99 higher)
Incontinence	100 (2 RCTs)	⊕○○○ Very low^a^, ^b^, ^d^	–	The mean incontinence was −0.14	MD 0.14 lower (1.15 lower to 0.86 higher)
Response rate	71 (1 RCT)	⊕⊕○○ Low^a^, ^d^	RR 1.30 (1.01 to 1.67)	686 per 1,000	891 more per 1,000 (693 more to 1,000 more)

^*^The risk in the intervention group (and its 95% confidence interval) is based on the assumed risk in the comparison group and the relative effect of the intervention (and its 95% CI).

CI, confidence interval; MD, mean difference; SMD, standardized mean difference.

^a^Downgraded by one level for study limitation: no limitation or serious of limitation.

^b^Downgraded by one level: high heterogeneity.

^c^Downgraded by one level for imprecision: confidence interval crossed assumed threshold of minimal clinically important difference or effect size.

^d^Downgraded by one level for imprecision: small sample size.

^e^Downgraded by two levels for study limitation: serious of limitation or very serious limitation. GRADE Working Group grades of evidence. High certainty: We are very confident that the true effect lies close to that of the estimate of the effect. Moderate certainty: We are moderately confident in the effect estimate: The true effect is likely to be close to the estimate of the effect, but there is a possibility that it is substantially different. Low certainty: Our confidence in the effect estimate is limited: The true effect may be substantially different from the estimate of the effect. Very low certainty: We have very little confidence in the effect estimate: The true effect is likely to be substantially different from the estimate of effect.

## Discussion

In this review, the advantages and possibilities of the use of AT for the treatment of OAB were identified. In the AT vs. sham AT comparison, significant effects in reducing OABSS and improving the response rate were noted for AT with very low certainty of evidence (CoE). Moreover, AT had a more favorable effect on reducing urinary frequency than sham AT with a low CoE. No significant differences in reducing urinary incontinence with a very low CoE were noted. However, AT exhibited effects equivalent to anticholinergic conventional drug therapy for reducing OABSS, urinary frequency, and urinary incontinence with a very low CoE. AT also had an equivalent effect as drug therapy for enhancing the response rate with a low CoE. Furthermore, the incidence of adverse effects was significantly lower with ATs compared with drug therapy with a low CoE. The combination of AT with drug therapy had a more favorable effect on reducing OABSS than drug therapy alone with a very low CoE. For reducing urinary frequency and incontinence, the combination of AT with drug therapy had a more favorable effect than drug therapy alone with a low CoE. Moreover, the combination of AT with drug therapy had a more favorable effect on enhancing the response rate than drug therapy alone with a low CoE. In the comparison of AT plus usual care with usual care, the AT plus usual care exhibited equivalent effects on reducing urinary frequency and incontinence with a very low CoE. However, regarding the response rate, the combination of AT and usual care had a more favorable effect with a very low CoE.

Our review aimed to evaluate and complete the evidence from recent RCTs of AT for the treatment of patients with OAB. Compared with two previous systematic reviews (46, 47), we identified 18 new RCTs (17, 18, 24, 27–31, 33–39, 43–45) and successfully assessed the evidence for therapy. The results of our review are different from those of the two previously published reviews. One previous review (47) showed that AT may be beneficial for reducing micturition, incontinence, and nocturia episodes, whereas the other review (46) failed to report favorable effects of AT for reducing symptoms of OAB compared with several types of controls. When we examined the results of AT for OAB symptoms, our results showed beneficial effects of AT compared with sham AT. However, one review did not show significant effects of AT compared with sham AT (47). Only two RCTs were included in the meta-analysis of electroacupuncture (EA) vs. sham EA, and EA had no favorable effect on reducing urinary frequency, urgency, or incontinence compared with sham EA. EA showed a favorable effect on decreasing nocturia. Another study (46) performed a meta-analysis of AT vs. sham AT on reducing OABSS based on two RCTs and did not show a significant effect. Instituting an appropriate sham AT condition is always a difficult factor in designing a study to determine the effects of AT. OAB affects the psychology of an individual, and sham AT for OAB was previously reported to produce a placebo effect in ~33–56% of participants (48). Emmons and Otto postulated a 40% placebo effect and a 59% treatment effect and explained that larger RCTs are needed to statistically show the effects of AT (16). Thus, it seems that the effect of sham AT, which has not been identified in previous reviews, appeared in this review, which includes more studies. However, since the size of the effect is not large, more studies are needed to collect the data. More consideration is needed to establish an appropriate sham AT to demonstrate the effect of AT appropriately.

Based on our assessment, the risk of bias is high in each of the included studies, potentially leading to false positives. Regarding performance bias, 23 studies had a high risk of bias based on the difference between intervention and control as well as AT treatment or drug administration. Although AT has to penetrate the skin and requires time for retention, medication is taken orally as prescribed. Therefore, blinding is difficult because patients can easily distinguish whether they are receiving AT or taking medications. Of the RCTs included in this study, there were no studies using AT and sham AT or drug and placebo drugs interchangeably. Moreover, AT could not be blinded to the performer (18, 23–45). Additional independent studies in different countries are required to determine the generalizability of these results given that 26 studies were conducted in China (17, 19–28, 30–42, 44, 45).

This review has some limitations. First, many included RCTs had an unclear risk of bias given that these studies did not report particular details. Second, despite the large number of RCTs, the outcomes were very diverse, so a large-scale meta-analysis could not be performed. Therefore, the level of evidence is mostly low or very low. Third, the frequency of AT intervention ranged from 2 per day to < 1 per week. Fourth, the suitable design of a sham AT condition remains a difficult problem.

Future studies on OAB treatment with AT should report their study design in more detail to obtain a high level of evidence. Moreover, if OABSS, response rate, adverse effects, and quality of life scores are commonly reported in these studies, we can obtain results with a larger population, definitively demonstrate the advantages of AT, and achieve a higher level of consensus. Studies should also be performed that could inform AT guidelines regarding acupoints, retention times, frequency, and treatment period for the application of AT for OAB in the clinical field.

In conclusion, AT had more favorable effects than sham AT in reducing OAB symptoms. AT showed equivalent effects as anticholinergic drugs with fewer AEs. The combination of AT and drug therapy had more favorable effects on OAB than drug therapy alone. However, the level of evidence is low due to the high risk of bias and the small sample size. Future well-designed research is needed to obtain a higher level of evidence to apply AT for the treatment of OAB.

## Data availability statement

The original contributions presented in the study are included in the article/Supplementary material, further inquiries can be directed to the corresponding authors.

## Author contributions

J-JL and J-IK: conceptualization. J-JL and J-WH: data curation and resources. J-JL, J-WH, MSL, and J-IK: formal analysis and writing—original draft. J-JL, J-WH, JHJ, and T-YC: investigation. J-JL, J-WH, and MSL: methodology. MSL and J-IK: project administration and supervision. J-WH and J-IK: software. JHJ and T-YC: writing—review and editing. All authors have read and approved the final manuscript.
