# Supplementary material for: Acupuncture for the treatment of overactive bladder: A systematic review and meta-analysis
Source: Front Neurol. 2023 Jan 12;13:985288. doi: 10.3389/fneur.2022.985288 (PMC9878288; doi:10.3389/fneur.2022.985288)
Supplement: Supplementary file 1 [file Data_Sheet_1.PDF]

Supplement 1. Search strategies

MEDLINE

1. exp overactive bladder/
2. Urinary Bladder, Overactive/
3. (urge incontinence or urinary incontinence). tw.
4. detrusor instability/
5. overactive detrusor/
6. (urinary urgency or urinary frequency).tw.
7. irritable bladder/
8. detrusor overactivity/
9. #1 OR #2 OR #3 OR #4 OR #5 OR #6 OR #7 OR #8
10. acupuncture/
11. exp acupuncture therapy/
12. electroacupuncture/
13. meridians/
14. acupuncture points/
15. acupuncture.tw.
16. (electroacupuncture or electro-acupuncture).tw.
17. acupoints.tw.
18. ((meridian or non-meridian or trigger) point).tw.
19. #10 OR #11 OR #12 OR #13 OR #14 OR # 15 OR #16 OR #17 OR #18
20. randomized controlled trial.pt.
21. controlled clinical trial.pt.
22. randomized.tiab.
23. placebo.tw.
24. clinical trials as topic.mh.
25. randomly.tiab.
26. trial.ti.
27. (crossover or cross-over or cross over).tw.
28. #20 OR #21 OR #22 OR #23 OR #24 OR #25 OR #26 OR #27

29. animals NOT humans.mh.

30. #28 NOT #29

31. # 9 AND #19 AND #30

CENTRAL

1. MeSH descriptor : [Urinary incontinence] explode all trees
2. MeSH descriptor : [Urge incontinence] explode all trees
3. Urinary frequency OR Urinary urgency: ti,ab,kw(Word variations have been searched)
4. Overactive bladder: ti,ab,kw(Word variations have been searched)
5. Irritable bladder: ti,ab,kw(Word variations have been searched)
6. Detrusor instability: ti ab kw(Word variations have been searched)
7. (overactive or overactivity or irritable or instability) near/5 (bladder* or detrusor*): ti, ab, kw(Word variations have been searched)
8. #1 OR #2 OR #3 OR #4 OR #5 OR #6 OR #7
9. acupuncture OR 'acupuncture therapy' OR electroacupuncture OR 'acupuncture points' OR electro-acupuncture OR acupoint* OR meridian* : ti, ab, kw (Word variations have been searched)
10. #8 and #9

EMBASE

1. 'Overactive bladder'/exp
2. 'irritable bladder'/exp
3. 'Detrusor instability'/exp
4. 'Urinary incontinence'/exp
5. 'Urinary frequency'/exp
6. ((overactive OR overactivity OR irritable OR instability) NEAR/5 (bladder* or detrusor*)): ab, ti
7. #1 OR #2 OR #3 OR #4 OR #5 OR #6
8. acupuncture OR 'acupuncture therapy' OR electroacupuncture OR 'acupuncture points' OR electro-acupuncture OR acupoint* OR meridian*
9. #7 and #8

10. 'crossover procedure':de OR 'double-blind procedure':de OR 'randomized controlled trial':de OR 'single-blind procedure':de OR (random* OR factorial* OR crossover* OR cross NEXT/1 over* OR placebo* OR doubl* NEAR/1 blind* OR singl* NEAR/1 blind* OR assign* OR allocat* OR volunteer*)

11. #9 and #10

AMED

1. overactive bladder : TX
2. detrusor instability : ab, ti
3. overactive detrusor : ab, ti
4. urinary urgency : ab, ti
5. urinary frequency : ab, ti
6. irritable bladder : ab, ti
7. detrusor overactivity : ab. ti
8. #1 OR #2 OR #3 OR #4 OR #5 OR #6 OR #7 OR #8
9. acupuncture OR ‘acupuncture therapy’ OR electroacupuncture OR ‘acupuncture points’ OR electro-acupuncture OR acupoint OR meridian
10. # 9 AND #10

CNKI

1 SU=('膀胱过度活动症'+'膀胱烦躁'+'逼尿肌不稳定性'+'尿频'+'过度活动+’) AND SU=(机) AND SU=('针')

2 AB=('膀胱过度活动症'+'膀胱烦躁'+'逼尿肌不稳定性'+'尿频'+'过度活动+’) AND AB=(机) AND AB=('针')

3 TI=('膀胱过度活动症'+'膀胱烦躁'+'逼尿肌不稳定性'+'尿频'+'过度活动 +’) AND TI=(机) AND TI=('针')

Wanfang

1 SU=('膀胱过度活动症'+'膀胱烦躁'+'逼尿肌不稳定性'+'尿频'+'过度活动

+'   ) AND SU=(机) AND SU=('针')

2 AB=('膀胱过度活动症'+'膀胱烦躁'+'逼尿肌不稳定性'+'尿频'+'过度活动

+'   ) AND AB=(机) AND AB=('针')

3 TI=('膀胱过度活动症'+'膀胱烦躁'+'逼尿肌不稳定性'+'尿频'+'过度活动   +'

) AND TI=(机) AND TI=('针')

VIP

1 SU=('膀胱过度活动症'+'膀胱烦躁'+'逼尿肌不稳定性'+'尿频'+'过度活动

+'   ) AND SU=(机) AND SU=('针')

2 AB=('膀胱过度活动症'+'膀胱烦躁'+'逼尿肌不稳定性'+'尿频'+'过度活动

+'   ) AND AB=(机) AND AB=('针')

3 TI=('膀胱过度活动症'+'膀胱烦躁'+'逼尿肌不稳定性'+'尿频'+'过度活动   +'

) AND TI=(机) AND TI=('针')

Koreamed

1. (과민성 방광 OR 절박뇨 OR 절박성 요실금 OR 배뇨근 불안정 OR 'overactive bladder' OR 'Irritable bladder' OR 'Urinary frequency' OR 'Urinary incontinence' OR 'Detrusor instability') AND (침 OR acupuncture)

KTKP

1. (과민성 방광 OR 절박뇨 OR 절박성 요실금 OR 배뇨근 불안정 OR 'overactive

bladder' OR 'Irritable bladder' OR 'Urinary frequency' OR 'Urinary incontinence' OR 'Detrusor instability') AND (침 OR acupuncture)

Dbpia

1. (과민성 방광 OR 절박뇨 OR 절박성 요실금 OR 배뇨근 불안정 OR 'overactive bladder' OR 'Irritable bladder' OR 'Urinary frequency' OR 'Urinary incontinence' OR 'Detrusor instability') AND (침 OR acupuncture)

RISS

1. (과민성 방광 OR 절박뇨 OR 절박성 요실금 OR 배뇨근 불안정 OR 'overactive bladder' OR 'Irritable bladder' OR 'Urinary frequency' OR 'Urinary incontinence' OR 'Detrusor instability') AND (침 OR acupuncture)

KISS

1. (과민성 방광 OR 절박뇨 OR 절박성 요실금 OR 배뇨근 불안정 OR 'overactive bladder' OR 'Irritable bladder' OR 'Urinary frequency' OR 'Urinary incontinence' OR 'Detrusor instability') AND (침 OR acupuncture)

Supplement 2. Descriptions of the AT interventions according to the revised STRICTA

First Author (year) (Ref)	Acupunctur e Rationale (reason)	Details of needling							Treatment Regime (Total session)	Other components of treatment	Practitioner background	Control or Comparator intervention
		**Number of needle**	**Names of acupoints (Uni /Bilateral)**	**Depth of Insertion**	**Response sought**	**Needle stimulation**	**Needle retention time**	**Needle type**				
Lin (2020) (17)	TCM theory (Consensus)	13	BL32, BL23, SP6, BL39, KI3, BL28(B), CV4(U)	BL32, BL23, (1.2cun) KI3(0.8cun) SP6, BL39, BL28, CV4(1.0cun)	Reinforcing technique with the lifting and thrusting movement	manual	30min	D: 0.3mm; L: 25, 40, 75mm Huatuo, Suzhou, China	8W, 2/W (16sessions)	n.r.	Registered CM practitioner (at least 3y of clinical experience in AT practice)	Sham AT (Non needle insertion, Park sham device)
Aydoğmuş (2014) (18)	TCM theory (Consensus)	19	LI4, ST36, SP6 KI3, KI5, LR3 (B)YINGTANG DU22, CV4(U)	LI4, ST36(1cun) SP6(0.8cun) KI3,KI5(0.4cun), YINGTANG, LR3 (0.3cun) DU22 (0.2cun) CV4 (0.5cun)	de qi	manual	20min	D:0.25mm L:25mm	4W, 2/W (8sessions)	n.r.	Licensed& experienced AT	Drug Sham AT
Emmons (2005) (16)	TCM theory (reports and consensus)	7	SP6, BL39, BL28(B), CV4(U)	n.r.	de qi rotate clockwise	manual	20min	n.r.	4W, 1/W (4sessions)	n.r.	n.r.	Placebo AT GB31,ST36 BL12 (B) CV12(U)
Zhang (2015) (19)	TCM theory (n,r.)	6	BL32, BL33 BL34(B)	50mm	de qi	electrical	30min	D:0.3mm; L:75mm, Suzhou Jiangsu, China	6W, 5/W (30sessions)	n.r.	More than 15 y clinical experience	Sham EA
Yu (2015) (20)	TCM theory (National Standard of China)	4	BL33, BL35(B)	2-3 cun	de qi	Electrical	30min	D:0.30mm; L:3cun, Suzhou Medical Products Guang Co., Ltd.	4W. 3/W (12sessions)	n.r.	n.r.	Sham EA (Non needle insertion BL33,BL35(B))
Wang (2013a) (21)	TCM theory (previous publication)	16	CV3, CV4(U) BL23, LR10, SP9, ST28, BL29, BL28, BL32(unclear)	n.r.	n.r.	Manipulate for 1m strongly BL29, BL28, BL32, CV3 at every 10min	30min	D:0.32-0.38mm L:15-40mm, Suzhou Medical Supplies Factory	4W, 1/D (28sessions)	NA	n.r.	Drug
Wang (2018) (22)	TCM theory (n.r.)	10 (plus addition n.r.detail)	BL23, SP6, BL28, BL29, BL32 (unclear)	n.r.	n.r.	manual	30min	n.r.	4W, 6/W (24sessions)	n.r.	n.r.	Drug
Yuan (2015) (23)	TCM theory (n,r.)	7	SP6, SP9, KI3(B), CV4(U)	1-1.5cm	de qi	manual	20min	D:0.35mm;L:50 mm; Huatuo Suzhou, China	4W,1/W (4sessions)	NA	More than 10y	Drug
Wang (2011) (24)	TCM theory (n.r.)	12	CV3,CV6, CV4, GV20(U), BL28, BL23, SP6, BL32 (unclear)	n.r.	n.r.	manual	20min	n.r.	4W, 1/D (28sessions)	n.r.	n.r.	Drug
Yu (2011) (25)	TCM theory (n.r.)	8	BL23, BL28, ST36, SP6(B)	n.r.	n.r.	manual	n.r.	n.r.	2W, 2/D (28sessions)	n.r.	n.r.	Drug

Kelleher (1994) (26)	TCM theory (literature)	15	SP6, ST36, BL23, BL28(B), CV3/4(U) Two paravertebral lumbar, segmental points, four sacral, segmental points	A few mm	Minimal stimulation technique	manual	10min	D:36gauge L:30mm	6W,1/W (6sessions)	n.r.	Experienced acupuncturist	Drug
Zhu (2021a) (27)	Nerve stimulation (n.r.)	4	Percutaneously penetrate the pudendal nerve running area	80mm	Patient's feeling	Electrical	45 min	D:0.30mm L:100mm	28 days (12 sessions)	n.r.	n.r.	Drug
Zhu (2021b) (28)	TCM theory (n.r.)	9+4~8	Huán zhōng shàng, SP6, BL32, BL28 (unclear), CV3(U) Additional points : ST36(unclear), CV4, CV6(U) (spleen-kidney qi deficiency) or ST36, BL23, BL20 (unclear), CV4, GV4(U), (spleen-kidney yang deficiency)	Huán Zhōng Shàng (3-5cun)	Huán Zhōng Shàng (de qi)	Manual Hun Zhōng Shàng (Manipulate bird pecking technique) Electrical: others	30min (except Huán Zhōng shàng)	n.r.	2W, 1/D (14sessions)	Electromagnetic wave treatment	n.r.	Drug
Chen (2009) (29)	TCM theory (n.r.)	6	CV2, CV1(U) BL35, BL32 (unclear)	n.r.	de qi	Electrical	30min	n.r.	14D. 1/D (14sessions)	n.r.	n.r.	Drug
Su (2020) (30)	TCM theory (n.r.)	6	CV3, CV4(U), SP6(B)	CV3(13-25mm), CV4,SP6 (30mm)	de qi	manual	20 min	D:0.25mm L:40mm	12 weeks, 3/W (36 sessions)	n.r.	n.r.	Drug
Mao (2012) (31)	TCM theory (Consensus)	8-10	Use two sets of acupoints alternately 1 : CV3, CV4(U), ST36, SP9, SP6 (unclear) 2 : BL23, BL28 BL35, BL32, BL54 (unclear)	CV3,CV4, ST36,SP9, SP6 (1.5-3cm) BL23,BL2, BL54 (1-2cm) BL35,BL32 (0.8-1.5cm)	de qi	Manual. Manipulate 5min BL23, SP6	30min	D:n.r. L:1-1.5cun	36D, 1/D 10 continuous days and rest 3 days (30sessions)	Infrared at abdomen and lumbo-sacral region	n.r.	Drug
Li (2017) (32)	TCM theory (n.r.)	8+4~6 (add according to TCM classify-cation)	KI3, LR3, BL64, BL58(B)	KI3 (0.5-0.8 cun) LR3, BL64 (0.3-0.5 cun) BL58 (1.0-1.5 cun)	de qi	manual	40min, Manipul-ate at 20min	D:0.35mm; L:40mm; Andy Pharmaceutic al Co.	12W, 1/D (About 66 sessions, 10 days continuous and 1~2 day rest)	n.r.	n.r.	Drug
Xiong (2020) (33)	n.r.	n.r.	myofascial trigger point or near the upper, lower, left and right sides	n.r.	needle sensation transmit-ed to the bladder& perineum	Electrical	30 min	D:0.5mm L:100mm	12W (42sessions)	n.r.	Professional rehabilitation therapists	Drug
Chen (2019) (34)	TCM theory (previous article)	10	EA: BL33(B) MA: BL23, BL28, BL40, KI3(B)	BL33 (1-2cun) Others n.r.	BL33: Soreness of the sacrum or tightness radiating to the perineum Others:de qi	Electrical Plus Manual.	30min	n.r.	4W, 5/W (20sessions)	n.r.	n.r.	Drug
Zhao (2012) (35)	TCM theory (n.r.)	17	MA: BL23, BL28, (unclear) EA: CV4,CV6, CV3(U) SP6, SP9, BL31, BL32, BL33 (unclear)	BL23,BL28, SP6,SP9 (n.r.) CV4,CV6, CV3 (20~30mm) BL31,BL32, BL33 (1.5cun)	de qi	Electrical Plus Manual	20min	D:0.30mm; L:50mm Jianweis Sterile AT needles	30D. 1/D (30sessions)	n.r.	n.r.	Drug

Chen (2015) (36)	TCM theory (n.r.)	9+4(add according to TCM classification)	CV4(U), KI3, BL35, BL23, HT7(unclear)	CV4(0.5-1cun) KI3(0.5-0.8cun) BL35(1.5cun) BL23(0.8-1cun) HT7(0.3-0.5cun)	de qi	Electrical	30min	D:0.25mm L:40mm	14D, 1/D (14 sessions)	TDP lamp	n.r.	Drug
Wang (2013b) (37)	TCM theory (n.r.)	8	EA: BL23, BL35(B) MA: BL32, SP6(unclear)	n.r.	n.r.	Electrical Plus Manual	30min	D:28-gauge L:2-inch	3M, 6/W (about 72sessions)	n.r.	n.r.	Drug
Liao (2007) (38)	TCM theory (n.r.)	10	EA: BL33(B) MA: BL23, BL28, BL40, KI3(B)	BL33 (1~2cun) BL23, BL28, BL40, KI3 (n.r.)	de qi	Electrical Plus Manual	30min	n.r.	4W, 5/W (20sessions)	n.r.	n.r.	Drug
Hargreaves (2021) (39)	TCM theory (literature)	13	CV3, CV4 CV5 (U) ST25,SP6, SP9, KI3, KI7(B)	CV3, CV4, CV5, ST25 (1cun) SP6, SP9, KI3, KI7 (0.5cun)	de qi turning in place after 10 and 20 min of treatment	manual	30min	D:n.r. L:40mm (CV3,CV4, CV5,ST25) 25mm (SP6, SP9, KI3, KI7)	8W (6sessions)	n.r.	Pelvic health physiotherapist, with 6 y of AT experience	standard conservative treatment
Xie (2020) (40)	TCM theory (n.r.)	8-10	alternately AT 1.BL23, BL35, BL28, BL32, BL54(unclear) 2.CV3,CV4(U), ST36, GB34, SP6(unclear)	CV3, CV4 (15-30mm) BL23, BL28 (10-20mm) BL54(10-20mm) Others n.r.	Correspond-ing sensations appear in the bladder, urethra, and perineum	manual	30min	n.r.	2M, 1/D (60sessions)	Infrared at sacrum and lower abdomen	n.r.	behavioral intervention care
Li (2012) (41)	TCM theory (n.r.)	10	BL28, BL32 BL23, BL29 BL26(unclear)	n.r.	n.r.	Manual manipulate for 1min strong	10min	n.r.	5W. 6/W (30sessions)	n.r.	n.r.	behavioral intervention care
**Number of papers* (%)**	**25(96) (9 (35))**	**25 (96)**	**26 (100)**	**12 (46)**	**19 (73)**	**26 (100)**	**25 (96)**	**15 (58)**	**26 (100)**	**6 (23)**	**7 (27)**	**26 (100)**

* Number of papers adequately reporting and their percentage in total. AT: acupuncture; CM: Chinese medicin
